# The Relationship between Fractional Flow Reserve, Platelet Reactivity and Platelet Leukocyte Complexes in Stable Coronary Artery Disease

**DOI:** 10.1371/journal.pone.0083198

**Published:** 2013-12-31

**Authors:** Jan-Willem E. M. Sels, Bert Rutten, Thijs C. van Holten, Marieke A. K. Hillaert, Johannes Waltenberger, Nico H. J. Pijls, Gerard Pasterkamp, Philip G. de Groot, Mark Roest

**Affiliations:** 1 Department of Cardiology, Catharina Hospital Eindhoven, Eindhoven, The Netherlands; 2 Department of Clinical Chemistry and Hematology, University Medical Center Utrecht, Utrecht, The Netherlands; 3 Laboratory of Experimental Cardiology, University Medical Center Utrecht, Utrecht, The Netherlands; 4 Department of Biomedical Engineering, Eindhoven University of Technology, Eindhoven, The Netherlands; 5 Department of Cardiology, University Medical Center Utrecht, Utrecht, The Netherlands; 6 Department of Cardiology, Maastricht University Medical Center, Maastricht. The Netherlands; 7 Department of Cardiovascular Medicine, University of Muenster, Muenster, Germany; King's College London School of Medicine, United Kingdom

## Abstract

**Background:**

The presence of stenoses that significantly impair blood flow and cause myocardial ischemia negatively affects prognosis of patients with stable coronary artery disease. Altered platelet reactivity has been associated with impaired prognosis of stable coronary artery disease. Platelets are activated and form complexes with leukocytes in response to microshear gradients caused by friction forces on the arterial wall or flow separation. We hypothesized that the presence of significantly flow-limiting stenoses is associated with altered platelet reactivity and formation of platelet-leukocyte complexes.

**Methods:**

One hundred patients with stable angina were studied. Hemodynamic significance of all coronary stenoses was assessed with Fractional Flow Reserve (FFR). Patients were classified FFR-positive (at least one lesion with FFR≤0.75) or FFR-negative (all lesions FFR>0.80). Whole blood samples were stimulated with increasing concentrations of ADP, TRAP, CRP and Iloprost with substimulatory ADP. Expression of P-selectin as platelet activation marker and platelet–leukocyte complexes were measured by flowcytometry. Patients were stratified on clopidogrel use. FFR positive and negative patient groups were compared on platelet reactivity and platelet-leukocyte complexes.

**Results:**

Platelet reactivity between FFR-positive patients and FFR-negative patients did not differ. A significantly lower percentage of circulating platelet-neutrophil complexes in FFR-positive patients and a similar non-significant decrease in percentage of circulating platelet-monocyte complexes in FFR-positive patients was observed.

**Conclusion:**

The presence of hemodynamically significant coronary stenoses does not alter platelet reactivity but is associated with reduced platelet-neutrophil complexes in peripheral blood of patients with stable coronary artery disease.

## Introduction

For patients with stable coronary artery disease, the presence and extent of myocardial ischemia is the most important prognostic factor for myocardial infarction and death [1]; [2]. On the other hand, patients who have coronary artery stenoses which do not significantly obstruct blood flow and consequently do not cause ischemia have a good prognosis, annual event rates being lower than 1% [3]. The biological mechanisms that mediate the increased risk in patients with inducible myocardial ischemia are not clear. Increased platelet reactivity is associated with increased risk of myocardial infarction (MI) in patients with stable coronary artery disease [4] and antithrombotic therapy has been shown to be effective in reducing the risk of future MI [5]. Furthermore, variation in response to antithrombotic therapy is associated with increased occurrence of atherothrombotic events in patients treated with percutaneous intervention [6]; [7]. Increased levels of platelet-monocyte complexes and increased platelet reactivity have been found in peripheral blood of patients with stable coronary artery disease compared to healthy control subjects [8] acute coronary syndromes [9]–[11] and ischemic stroke [12]–[14]. Platelets are functionally affected by conditions of high shear stress [15]; [16] and platelets form larger aggregates in response to increasing microshear gradients, independent of soluble agonists [17]. Previous reports have shown that lesion severity and calculated shear stress correlate with increased platelet-monocyte complexes distal to a stenosis and further in the coronary sinus, as compared with samples from the proximal coronary artery [18]. Also, experimental evidence suggests myocardial ischemia itself as a factor in platelet behavior, by secretion of proaggregratory substances [19]. Apart from thrombosis, it has become increasingly clear that platelets are actively involved in all stages of atherosclerosis. Platelets have been shown to interact with both endothelial cells as well as circulating leukocytes to promote atherogenesis [20]. Alpha granule fusion with the platelet membrane causes exposure of P-selectin which by interaction with P-selectin glycoprotein ligand – 1 (PSGL-1) mediates the formation of inflammatory platelet-leukocyte complexes. This facilitates a leukocyte influx into the endothelium, thereby presumably assisting in lesion development [21]. Fractional Flow Reserve (FFR) is an invasive lesion-specific index of myocardial ischemia due to epicardial coronary stenosis. FFR measures a lesion's ability to cause myocardial ischemia by measuring the pressure gradient across a stenosis during maximum induced hyperemia [22]. Moreover, patients with FFR-positive lesions benefit from revascularization and medical treatment of FFR-positive lesions is inferior to revascularization [23]. We hypothesized that in patients with stable coronary artery disease, the presence of ischemia-causing, flow-limiting coronary lesions, as measured by FFR is associated with altered platelet reactivity. Furthermore, we hypothesized that the presence of hemodynamically significant coronary lesions is associated with altered fractions of platelet-leukocyte complexes (PLCs).

## Methods

### Interventional procedure

The study was approved by the local ethics committee of all participating centers (Catharina Hospital Eindhoven, Utrecht University Medical Center, Maastricht University Medical Center, Leiden University Medical Center) and all patients gave written informed consent for participation prior to coronary angiography.

One hundred sixteen patients with stable angina, who were referred for angiography on basis of symptoms suggesting myocardial ischemia and/or evidence of ischemia on non-invasive testing, underwent FFR measurement were included from 3 Dutch hospitals: Catharina Hospital Eindhoven, University Medical Center Utrecht and the Maastricht University Medical Center. All patients were referred for angiography on basis of symptoms suggesting myocardial ischemia and/or evidence of ischemia on non-invasive testing. Exclusion criteria were active inflammatory state, auto-immune disease or malignancy. Fractional Flow Reserve of all coronary stenoses was performed according to established standard practice under conditions of maximal hyperemia. Evidence of myocardial ischemia was defined by the presence of at least one coronary stenosis with FFR≤0.75. Conversely ischemia was considered absent if none of the measured lesions had an FFR≤0.80. These cut off values have been extensively validated [24]. To clearly demarcate the presence or absence of ischemia, we did not include 16 patients with intermediate FFR-values of 0.76 to 0.80, leaving 100 patients for analysis. Decision on treatment of the coronary lesions was left at the discretion of the operator. Depending on whether PCI was expected to be performed on the basis of a previously performed angiography, patients received a loading dose of clopidogrel 600 mg on the day before the procedure, according to local protocol.

### Laboratory methods

Laboratory tests were conducted in three medical centers. In order to prevent variation between the individual centers, all materials were centrally ordered and disturbed from the university medical center in Utrecht and each center was equipped with a FC500 flow cytometer (Beckman Coulter) that was centrally calibrated at the university medical center in Leiden.

### Blood collection

Blood was collected from the arterial sheath into 3.2% tri-sodium citrate tubes (Greiner Bio-One), before administration of intravenous anticoagulants. All samples were processed directly after collection to prevent any potential blood storage effects.

### Platelet reactivity assay

Platelet reactivity was determined with agonist concentration series of adenosine diphosphate (ADP; P2Y_12_ receptor agonist), cross linked collagen related peptide (CRP-XL; GPVI receptor agonist), thrombin receptor activating peptide (TRAP; PAR-1 receptor agonist), and iloprost (IP receptor agonist) with substimulatory ADP. Serial dilutions of ADP (125 µM, 31.25 µM, 7.8 µM, 1.95 µM, 488 nM, 122 nM, 31 nM, 8 nM) were prepared in 50 µL HEPES buffered saline (HBS; 10 mM HEPES, 150 mM NaCl, 1 mM MgSO4, 5 mM KCl, pH 7.4), with 2 µL phycoerythrin (PE) labeled mouse anti-human P-selectin antibodies (BD Biosciences, Breda, the Netherlands) and 2 µL fluorescein isothiocyanate (FITC) labeled mouse anti-human GPIb antibodies (BD Biosciences, Breda, the Netherlands). Similarly, serial dilutions of CRP-XL (2.5 µgr/mL, 625 ng/mL, 156.3 ng/mL, 39.1 ng/mL, 9.8 ng/mL, 2.4 ng/mL, 600 pg/mL, 153 pg/mL), TRAP (625 µM, 156.3 µM, 39.1 µM, 9.8 µM, 2.4 µM, 610 nM, 153 nM, 38n M), and iloprost (1250 ng/mL, 312.5 ng/mL, 78 ng/mL, 19.5 ng/mL, 4.9 ng/mL, 1.2 ng/mL, 0.31 ng/mL, 0.076 ng/mL) with 5 µM ADP were prepared in 50 µL HBS with 2 µL mouse anti-human P-selectin antibodies and 2 µL mouse anti human GPIb antibodies. The platelet reactivity assay was initiated by adding 5 µL whole blood to each sample of serial dilutions. Samples were incubated for 20 minutes and subsequently added with 500 µL fixative (0.2% formaldehyde and 0.9% NaCl). All samples were analyzed on a FC 500 flow cytometer (Beckman Coulter, FL, USA) on the same day of processing. Data acquisition was performed with the SXP SYSTEM software (Beckman Coulter, FL, USA) Single platelets were gated based on FITC signal intensity. The median fluorescence intensity (MFI) for PE was measured. Dose-response graphs for P-selectin expression were constructed and the maximum response and area under the curves (AUC) in arbitrary units was calculated.

### Platelet-leukocyte complex assay

For assessment of platelet-leukocyte complexes, 50 µl of citrate anticoagulated whole blood was diluted with 45 ul phosphate buffered saline (PBS) and platelets were labeled by incubation with 5 µl FITC labelled mouse anti human GPIb antibodies (BD Biosciences) for 30 minutes at room temperature. Triplicate samples were fixed for 10 minutes with 80 µl of Optilyse B (Beckman Coulter), containing 3.4% paraformaldehyde, after which hypotonic red blood cell lysis was achieved by the addition of 820 µl of demineralized water. Monocytes and neutrophils were identified by forward and sideward scatter gating. Platelet – monocyte complexes (PMCs) and platelet – neutrophil complexes (PNCs) were determined by calculating the percentage of cells in these gates that were positive for the platelet marker GPIb. All samples were analyzed on a FC 500 flow cytometer (Beckman Coulter, FL, USA) on the same day of processing. Fifteen thousand cells were counted, and data acquisition was performed with the SXP SYSTEM software (Beckman Coulter, FL, USA).

### Statistical analysis

Comparison of categorical variables was done using Chi square testing,while continuous variables were compared using the Student T or Mann Whitney test as appropriate. A p-value <0.05 was considered statistically significant. We performed collinearity analysis and used the variance inflation factor (VIF), because previous research has demonstrated inhibition of clopidogrel on both platelet reactivity and platelet leukocyte complex formation [25]; [26]. Collinearity analysis of all independent variables showed significant collinearity of clopidogrel use and FFR (tolerance 0.037, VIF 27.4). To omit a confounding effect of clopidogrel use, we therefore stratified our patients according to clopidogrel use (either chronic or loading dose) yielding 4 groups: FFR-negative without clopidogrel (25 patients), FFR-positive without clopidogrel (9 patients), FFR-negative with clopidogrel (18 patients) and FFR-positive with clopidogrel (48 patients). All statistical analysis were performed using SPSS 18 (SPSS inc, Chicago, Ill, USA).

## Results

### Baseline characteristics

The baseline characteristics of the included patients are presented in table 1.

**Table 1:** Baseline characteristics of FFR-positive and FFR-negative patients, stratified on clopidogrel usage.

	FFR negative Clopidogrel -	FFR positive Clopidogrel –	*p-value	FFR negative Clopidogrel +	FFR positive Clopidogrel +	*p-value
	N = 25	N = 9		N = 18	N = 48	
Age (mean±SD)	62.7±8.4	64.5±8.3	0.59	63.6±11.7	61.3±10.0	0.46
Sex – Male (%)	15 (60)	6 (66.7)	0.73	12 (66.7)	30 (62.5)	0.76
Previous MI	5 (20)	3 (33.3)	0.43	8 (44.4)	10 (20.8)	0.06
Previous PCI	7 (28)	3 (33.3)	0.77	11 (61.1)	17 (35.4)	0.07
Previous CABG	3 (12)	0 (0)	0.29	1 (5.6)	1 (2.1)	0.56
DM	4 (16)	2 (22.2)	0.69	1 (5.6)	11 (22.9)	0.11
Hypertension	11 (44)	3 (33.3)	0.26	8 (44.4)	24 (50)	0.70
Hyperlipidemia	12 (48)	4 (44.4)	0.71	8 (44.4)	25 (25.1)	0.60
Smoking	5 (20)	2 (22.2)	0.89	9 (50)	11 (22.9)	**0.03**
Family history of CAD	14 (56)	5 (55.6)	0.98	9 (50)	37 (77.1)	**0.03**
ASA	22 (88)	9 (100)	0.08	15 (83.3)	44 (91.7)	0.41
B-blockers	12 (48)	8 (88.9)	**0.03**	11 (66.1)	42 (87.5)	**0.02**
Statins	21 (84)	8 (88.9)	0.73	17 (94.4)	42 (87.5)	0.35
ACE-inhibitors	6 (24)	3 (33.3)	0.60	7 (38.9)	13 (27.1)	0.39
Oral anticoagulation	2 (8)	0 (0)	0.40	0 (0)	3 (6.3)	0.29
Non-significant	2 (8)	0 (0)	1.0	1 (5.6)	0 (0)	0.27
1 – vessel disease	16 (64)	3 (33.3)	0.13	7 (38.9)	28 (58.3)	0.18
2 – vessel disease	5 (20)	2 (22.2)	1.0	6 (33.3)	16 (33.3)	1.0
3 – vessel disease	2 (8)	4 (44.4)	0.03	4 (22.2)	4 (8.3)	0.2
						

Continuous values are presented as means ± SD. Categorical values are presented as number (percentages). Non-significant indicates no lesion with stenosis >50%.

*Significant p-values are printed bold. MI = myocardial infarction, PCI =  percutaneous coronary intervention, CABG =  coronary artery bypass grafting, DM =  diabetes mellitus, CAD =  coronary artery disease.

As shown in table 1, baseline characteristics in general did not differ significantly between FFR-negative and FFR-positive patients, except for smoking and a family history of coronary artery disease, which were more prevalent in the FFR negative patients that were treated with clopidogrel. Use of Beta blockers was more prevalent in FFR-positive patients than in FFR-negative patients. The mean lowest FFR measured per patient was 0.55±0.14 in FFR-positive patients versus 0.87±0.04 indicating a clear distinction between ischemic and non-ischemic coronary artery disease patients.

### Platelet reactivity

**Figure 1:**
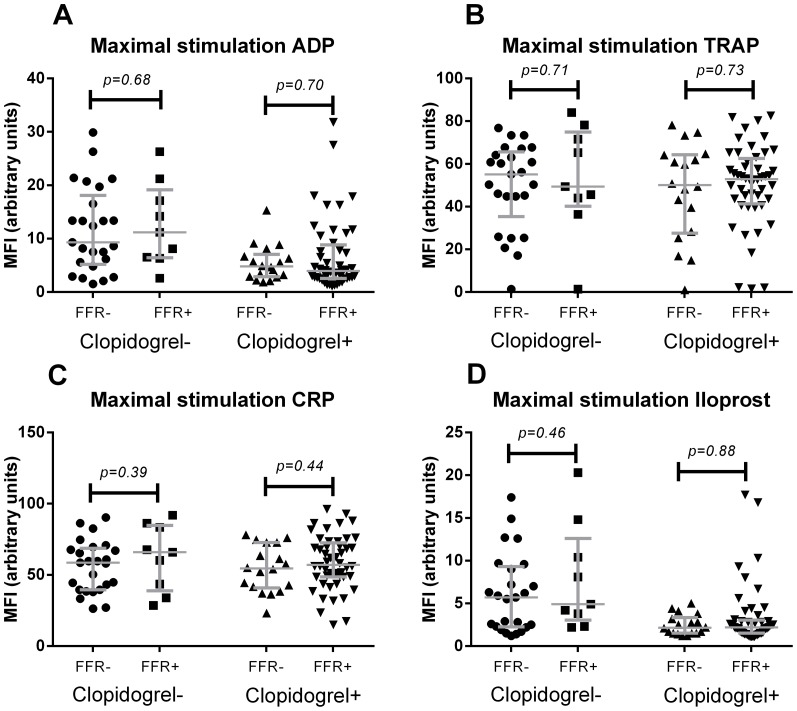
Maximal expression of P-selectin after stimulation with ADP (A), TRAP (B), CRP (C) and Iloprost with substimulatory ADP (D). Lines indicate median values with interquartile range.

**Figure 2:**
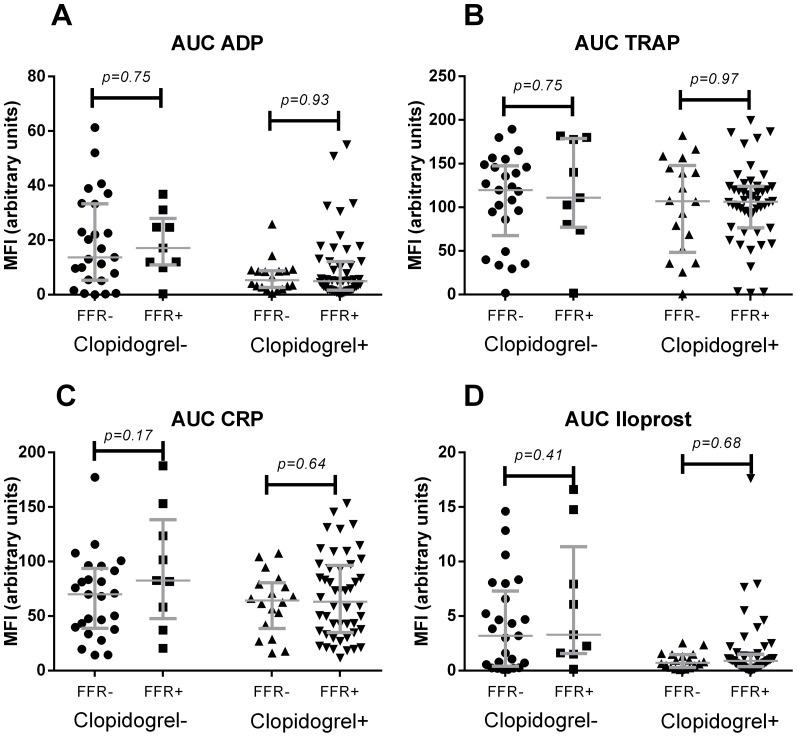
Cumulative expression of P-selectin after stimulation with ADP (A), TRAP (B), CRP (C) and Iloprost with substimulatory ADP (D). Lines indicate median values with interquartile range.

Platelet reactivity was determined by the maximal expression of P-selectin after stimulation and cumulative reactivity by the area under the curve (AUC) calculations determined from the dose-response curves. The use of clopidogrel resulted in a markedly lower response to ADP, and Iloprost with sub-optimal ADP stimulation for both the maximal response and the AUC. Clopidogrel was not able to significantly inhibit activation by TRAP or CRP-XL as was observed in both the maximal response and the AUC values (Figure 1 and 2). No significant difference was observed between FFR-positive and FFR-negative patients for all activation responses by either platelet reactivity measurements.

### Platelet-leukocyte complexes

**Figure 3:**
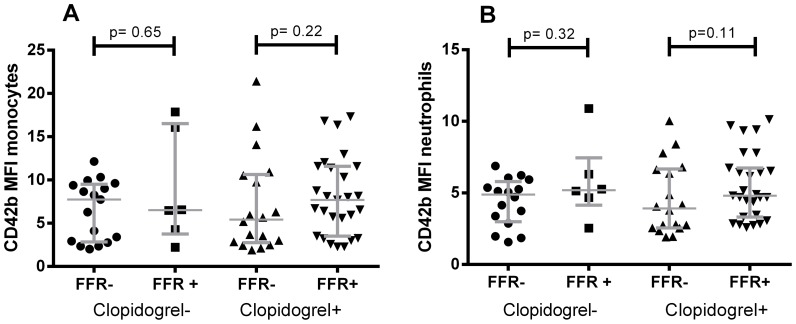
Platelets per monocyte (A) and neutrophil (B) measured by MFI of CD42b. Lines indicate median values with interquartile range.

**Figure 4:**
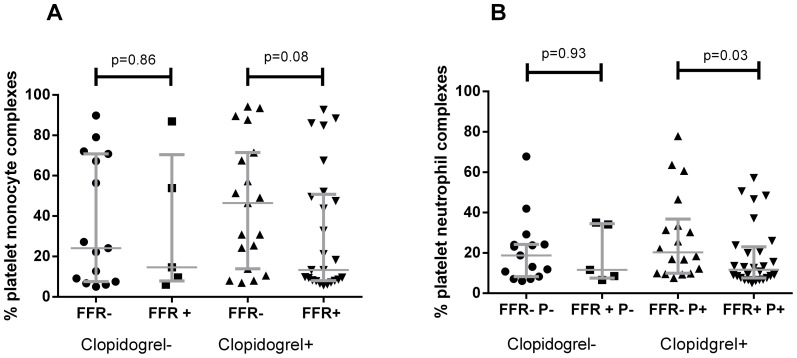
Percentages of platelet-monocyte (A) and platelet-neutrophile (B) complexes in FFR-negative and FFR-positive patients, identified by presence of platelet marker CD42b on monocytes and neutrophils. Lines indicate median values with interquartile range.

Percentages of platelet–monocyte complexes (PMCs) and platelet–neutrophil complexes (PNCs) in whole blood were available for 67 included patients. Patients were again stratified according to clopidogrel use and percentages of complexes were compared between FFR-positive and negative patients. The number of platelets per leukocyte, as measured by MFI of CD42b, did not significantly differ between groups(Figure 3). We observed no significant difference in percentage of PMCs or PNCs between the FFR-positive and FFR-negative patients that were not treated with clopidogrel, although comparison is hampered by the small sample size (n = 15 vs n = 5) (Figure 4A). There was a significant decrease in the percentage of PNCs (p = 0.03; Figure 4B) and a trend towards a decrease in the percentage of PMCs (p = 0.08; Figure 4A) in the clopidogrel treated FFR-positive patients compared to FFR-negative patients.

## Discussion

In this observational study we observed no differences in both maximal and cumulative in vitro platelet reactivity between patients with a positive FFR and a negative FFR. However, we did observe a significantly lower percentage PNCs in patients with a positive FFR compared to patients with negative FFR in the clopidogrel treated group (p = 0.03), and a trend towards lower percentages of PMCs in patients with a positive FFR compared to a negative FFR in the clopidogrel treated group (p = 0.08).

Previous clinical studies have shown diverging results with respect to the relationship between platelet reactivity, platelet-leukocyte complexes and coronary luminal obstruction or inducible myocardial ischemia. Increased systemic platelet reactivity was described in patients with documented coronary artery disease immediately after peak exercise [27]; [28], however no relationship with ischemia was found [27]. In critical limb ischemia, increased PMCs and expression of P-selectin was observed, implying an ischemic mechanism of platelet activation [29]. Conversely, other investigators found an inverse relationship between coronary obstruction and platelet reactivity [30], although all patients investigated had severe single-vessel disease. Others found experimental evidence that platelet reactivity might be reduced by ischemic pre-conditioning [31], which may point to the possibility of down regulation of platelet reactivity by repeated, short-acting bouts of ischemia as occurs in stable coronary disease. We found no evidence for this.

The paradoxical lower percentage of platelet-leukocyte complexes observed in the FFR-positive group may be explained by increased activation of leukocytes due to significantly flow-limiting stenoses [14] and subsequent increased clearance of the complexes from the circulation. Da Costa *et al* showed that attachment of monocytes to platelets leads to enhanced transmigration of monocytes into the subendothelium [32]. Also, Huo et al showed that the interaction of infused activated platelets with leukocytes resulted in increased adherence to the endothelium. Subsequent transmigration of the complexes led to absence of detectable levels of platelet-leukocyte complexes in a time frame of 3–4 hours [33]. Acute ischemic events, like myocardial and cerebral infarction cause a strong inflammatory response and tissue damage, and are associated increased levels of peripherally detectable leukocyte-platelet formation during or shortly after the ischemic event, as previously shown [9]–[14]. In contrast, inducible ischemia in stable coronary disease implies relatively short, reversible episodes of ischemia (during for example exercise) without permanent damage, which may transiently increase leukocyte-platelet complexes. PLCs may subsequently be cleared from the circulation by the above mentioned mechanisms, effectively leading to decreased levels when measured in stable, non-ischemic conditions. Besides lower systemic percentages of platelet-leukocyte complexes due to increased transmigration of these complexes, neutrophils have been shown to phagocytose activated platelets in vivo [34]. Hence, lower percentages of circulating neutrophils with platelets adhered to the cell membrane might reflect leukocyte populations that effectively phagocytosed activated platelets.

### Limitations

This study has several limitations. In our population, platelet reactivity and platelet–leukocyte complexes were assessed in stable, non-ischemic conditions, as opposed to the majority of previously mentioned research in which platelets and platelet – leukocyte complexes were mostly investigated during or shortly after ischemia or exercise. Thus, we cannot discount the possibility that acute changes in platelet reactivity occur during ischemic episodes, which subsequently disappear when ischemia is resolved, presumably by increased removal of formed PLCs. Another limitation is the fact that blood samples were drawn from the arterial sheath, which may potentially dilute any local effect caused by the FFR-positive lesion. Systemic blood sampling may not recognize local PLC formation in the coronary circulation, although previous research have also found differences in PLCs in systemic blood samples of patients with localized arterial pathology [9], [29].

In this observational study, patients were included before coronary angiography and FFR, which may account for differences in baseline characteristics. Importantly, use of clopidogrel before angiography differed significantly between FFR –positive and FFR-negative patient groups. The reason for this is that the majority of these patients were referred for PCI in which loading of clopidogrel is mandatory, in contrast to coronary angiography with FFR alone, in which this is not required. This introduces a bias between the FFR-positive and negative groups, since patients with severe lesions are more likely to be referred for PCI and thus be treated with clopidogrel upfront while at the same time these are more likely to have a FFR≤0.75. Given the observational nature of this study, however, we could not interfere with local regimens. We therefore chose to stratify patients, which resulted in relatively small groups and reduced statistical power. More precisely, the low sample sizes after stratification, although justified, prevented us from performing a meaningful multivariate analysis, especially since the data are non-normally distributed. Our findings therefore need further validation with larger sample sizes.

## References

[1] Hachamovitch R, Berman DS, Shaw LJ, Kiat H, Cohen I, et al (1998) Incremental prognostic value of myocardial perfusion single photon emission computed tomography for the prediction of cardiac death: differential stratification for risk of cardiac death and myocardial infarction. Circulation 97: 535–43. 9494023 10.1161/01.cir.97.6.535

[2] Iskandrian AS, Chae SC, Heo J, Stanberry CD, Wasserleben V, et al (1993) Independent and incremental prognostic value of exercise single-photon emission computed tomographic (SPECT) thallium imaging in coronary artery disease. J Am Coll Cardiol 22: 665–70. 8354796 10.1016/0735-1097(93)90174-y

[3] Pijls NH, van Schaardenburgh P, Manoharan G, Boersma E, Bech JW, et al (2007) Percutaneous coronary intervention of functionally nonsignificant stenosis: 5-year follow-up of the DEFER Study. J Am Coll Cardiol 49: 2105–11. 17531660 10.1016/j.jacc.2007.01.087

[4] Snoep JD, Roest M, Barendrecht AD, de Groot PG, Rosendaal FR, et al (2010) High platelet reactivity is associated with myocardial infarction in premenopausal women: a population-based case-control study. J Thromb Haemost 8: 906–13. 20128867 10.1111/j.1538-7836.2010.03786.x

[5] Baigent C, Blackwell L, Collins R, Emberson J, Godwin J, et al (2009) Aspirin in the primary and secondary prevention of vascular disease: collaborative meta-analysis of individual participant data from randomised trials. Lancet 373: 1849–60. 19482214 10.1016/S0140-6736(09)60503-1 PMC2715005

[6] Geisler T, Langer H, Wydymus M, Gohring K, Zuring C, et al (2006) Low response to clopidogrel is associated with cardiovascular outcome after coronary stent implantation. Eur Heart J 27: 2420–5. 17005534 10.1093/eurheartj/ehl275

[7] Braunwald E, Angiolillo D, Bates E, Berger PB, Bhatt D, et al (2008) The problem of persistent platelet activation in acute coronary syndromes and following percutaneous coronary intervention. Clin Cardiol 31: I17–I20. 18481817 10.1002/clc.20363 PMC6653003

[8] Furman MI, Benoit SE, Barnard MR, Valeri CR, Borbone ML, et al (1998) Increased platelet reactivity and circulating monocyte-platelet aggregates in patients with stable coronary artery disease. J Am Coll Cardiol 31: 352–8. 9462579 10.1016/s0735-1097(97)00510-x

[9] Sarma J, Laan CA, Alam S, Jha A, Fox KA, et al (2002) Increased platelet binding to circulating monocytes in acute coronary syndromes. Circulation 105: 2166–71. 11994250 10.1161/01.cir.0000015700.27754.6f

[10] Furman MI, Barnard MR, Krueger LA, Fox ML, Shilale EA, et al (2001) Circulating monocyte-platelet aggregates are an early marker of acute myocardial infarction. J Am Coll Cardiol 38: 1002–6. 11583872 10.1016/s0735-1097(01)01485-1

[11] Brambilla M, Camera M, Colnago D, Marenzi G, De Metrio M (2008) Tissue factor in patients with acute coronary syndromes: expression in platelets, leukocytes, and platelet-leukocyte aggregates. Arterioscler Thromb Vasc Biol 28: 947–53. 18292391 10.1161/ATVBAHA.107.161471

[12] Ishikawa T, Shimizu M, Kohara S, Takizawa S, Kitagawa Y, et al (2012) Appearance of WBC-platelet complex in acute ischemic stroke, predominantly in atherothrombotic infarction. J Atheroscler Thromb 19: 494–501. 22659534 10.5551/jat.10637

[13] McCabe DJ, Harrison P, Mackie IJ, Sidhu PS, Purdy G (2005) Increased platelet count and leucocyte-platelet complex formation in acute symptomatic compared with asymptomatic severe carotid stenosis. J Neurol Neurosurg Psychiatry 76: 1249–54. 16107361 10.1136/jnnp.2004.051003 PMC1739812

[14] McCabe DJ, Harrison P, Mackie IJ, Sidhu PS, Purdy G, et al (2004) Platelet degranulation and monocyte-platelet complex formation are increased in the acute and convalescent phases after ischaemic stroke or transient ischaemic attack. Br J Haematol 125: 777–87. 15180868 10.1111/j.1365-2141.2004.04983.x

[15] Kroll MH, Hellums JD, McIntire LV, Schafer AI, Moake JL (1996) Platelets and shear stress. Blood 88: 1525–41. 8781407

[16] Kroll MH, Hellums JD, Guo Z, Durante W, Razdan K, et al (1993) Protein kinase C is activated in platelets subjected to pathological shear stress. J Biol Chem 268: 3520–4. 8429027

[17] Nesbitt WS, Westein E, Tovar-Lopez FJ, Toulouei E, Mitchell A, et al (2009) A shear gradient-dependent platelet aggregation mechanism drives thrombus formation. Nat Med 15: 665–73. 19465929 10.1038/nm.1955

[18] Yong AS, Pennings GJ, Chang M, Hamzah A, Chung T, et al (2011) Intracoronary shear-related up-regulation of platelet P-selectin and platelet-monocyte aggregation despite the use of aspirin and clopidogrel. Blood 117: 11–20. 20876457 10.1182/blood-2010-04-278812

[19] Gurbel PA, Serebruany VL, Komjathy SF, Collins ME, Sane DC, et al (1995) Regional and Systemic Platelet Function Is Altered by Myocardial Ischemia-Reperfusion. J Thromb Thrombolysis 1: 187–94. 10603529 10.1007/BF01062577

[20] Gawaz M, Langer H, May AE (2005) Platelets in inflammation and atherogenesis. J Clin Invest 115: 3378–84. 16322783 10.1172/JCI27196 PMC1297269

[21] Linden MD, Jackson DE (2010) Platelets: pleiotropic roles in atherogenesis and atherothrombosis. Int J Biochem Cell Biol 42: 1762–6. 20673808 10.1016/j.biocel.2010.07.012

[22] Pijls NH, Sels JW (2012) Functional measurement of coronary stenosis. J Am Coll Cardiol 59: 1045–57. 22421298 10.1016/j.jacc.2011.09.077

[23] De Bruyne B, Pijls NH, Kalesan B, Barbato E, Tonino PA, et al (2012) Fractional Flow Reserve-Guided PCI versus Medical Therapy in Stable Coronary Disease. N Engl J Med 367: 991–1001. 22924638 10.1056/NEJMoa1205361

[24] De Bruyne B, Sarma J (2008) Fractional flow reserve: a review: invasive imaging. Heart 94: 949–59. 18552231 10.1136/hrt.2007.122838

[25] Klinkhardt U, Bauersachs R, Adams J, Graff J, Lindhoff-Last E, et al (2003) Clopidogrel but not aspirin reduces P-selectin expression and formation of platelet-leukocyte aggregates in patients with atherosclerotic vascular disease. Clin Pharmacol Ther 73: 232–41. 12621388 10.1067/mcp.2003.13

[26] Braun OO, Johnell M, Varenhorst C, James S, Brandt JT, et al (2008) Greater reduction of platelet activation markers and platelet-monocyte aggregates by prasugrel compared to clopidogrel in stable coronary artery disease. Thromb Haemost 100: 626–33. 18841285

[27] Andreotti F, Lanza GA, Sciahbasi A, Fischetti D, Sestito A, et al (2001) Low-grade exercise enhances platelet aggregability in patients with obstructive coronary disease independently of myocardial ischemia. Am J Cardiol 87: 16–20. 11137827 10.1016/s0002-9149(00)01265-0

[28] Wallen NH, Held C, Rehnqvist N, Hjemdahl P (1997) Effects of mental and physical stress on platelet function in patients with stable angina pectoris and healthy controls. Eur Heart J 18: 807–15. 9152651 10.1093/oxfordjournals.eurheartj.a015346

[29] Burdess A, Nimmo AF, Campbell N, Harding SA, Garden OJ, et al (2010) Perioperative platelet and monocyte activation in patients with critical limb ischemia. J Vasc Surg 52: 697–703. 20816321 10.1016/j.jvs.2010.04.024

[30] Milovanovic M, Fransson SG, Richter A, Jaremo P (2005) Inverse relationships between coronary blood flow obstruction and platelet reactivity in stable angina pectoris. Platelets 16: 211–3. 16011966 10.1080/09537100400016813

[31] Linden MD, Whittaker P, Frelinger AL III, Barnard MR, Michelson AD, et al (2006) Preconditioning ischemia attenuates molecular indices of platelet activation-aggregation. J Thromb Haemost 24: 2670–7. 10.1111/j.1538-7836.2006.02228.x 16995902

[32] da Costa MP, van den BN, Ulfman LH, Koenderman L, Hordijk PL, et al (2004) Platelet-monocyte complexes support monocyte adhesion to endothelium by enhancing secondary tethering and cluster formation. Arterioscler Thromb Vasc Biol 24: 193–9. 14615387 10.1161/01.ATV.0000106320.40933.E5

[33] Huo Y, Schober A, Forlow SB, Smith DF, Hyman MC, et al (2003) Circulating activated platelets exacerbate atherosclerosis in mice deficient in apolipoprotein E. Nat Med 9: 61–7. 12483207 10.1038/nm810

[34] Maugeri N, Rovere-Querini P, Evangelista V, Covino C, Capobianco A, et al (2009) Neutrophils phagocytose activated platelets in vivo: a phosphatidylserine, P-selectin, and {beta}2 integrin-dependent cell clearance program. Blood 113: 5254–65. 19264679 10.1182/blood-2008-09-180794

